# Genomics of Environmental *Salmonella*: Engaging Students in the Microbiology and Bioinformatics of Foodborne Pathogens

**DOI:** 10.3389/fmicb.2021.592422

**Published:** 2021-04-22

**Authors:** Noah A. Greenman, Sophie K. Jurgensen, Charles P. Holmes, Curtis J. Kapsak, Raechel E. Davis, William M. Maza, Desiree Edemba, Bethany A. Esser, Selena M. Hise, Tara N. Keen, Hunter G. Larson, Dominique J. Lockwood, Brian Wang, Joseph A. Harsh, James B. Herrick

**Affiliations:** ^1^Department of Biology, James Madison University, Harrisonburg, VA, United States; ^2^Center for Genome and Metagenome Studies, James Madison University, Harrisonburg, VA, United States

**Keywords:** *Salmonella*, course-based undergraduate research, pathogen reservoirs, bioinformatics, pathogen surveillance, environmental pathogens, public health microbiology, genomic epidemiology

## Abstract

We have developed and implemented an undergraduate microbiology course in which students isolate, characterize, and perform whole genome assembly and analysis of *Salmonella enterica* from stream sediments and poultry litter. In the development of the course and over three semesters, successive teams of undergraduate students collected field samples and performed enrichment and isolation techniques specific for the detection of *S. enterica*. Eighty-eight strains were confirmed using standard microbiological methods and PCR of the *invA* gene. The isolates’ genomes were Illumina-sequenced by the Center for Food Safety and Applied Nutrition at the FDA and the Virginia state Division of Consolidated Laboratory Services as part of the GenomeTrakr program. Students used GalaxyTrakr and other web- and non-web-based platforms and tools to perform quality control on raw and assembled sequence data, assemble, and annotate genomes, identify antimicrobial resistance and virulence genes, putative plasmids, and other mobile genetic elements. Strains with putative plasmid-borne antimicrobial resistance genes were further sequenced by students in our research lab using the Oxford Nanopore MinION^TM^ platform. Strains of *Salmonella* that were isolated include human infectious serotypes such as Typhimurium and Infantis. Over 31 of the isolates possessed antibiotic resistance genes, some of which were located on large, multidrug resistance plasmids. Plasmid pHJ-38, identified in a Typhimurium isolate, is an apparently self-transmissible 183 kb IncA/C2 plasmid that possesses multiple antimicrobial resistance and heavy-metal resistance genes. Plasmid pFHS-02, identified in an Infantis isolate, is an apparently self-transmissible 303 kb IncF1B plasmid that also possesses numerous heavy-metal and antimicrobial resistance genes. Using direct and indirect measures to assess student outcomes, results indicate that course participation contributed to cognitive gains in relevant content knowledge and research skills such as field sampling, molecular techniques, and computational analysis. Furthermore, participants self-reported a deeper interest in scientific research and careers as well as psychosocial outcomes (e.g., sense of belonging and self-efficacy) commonly associated with student success and persistence in STEM. Overall, this course provided a powerful combination of field, wet lab, and computational biology experiences for students, while also providing data potentially useful in pathogen surveillance, epidemiological tracking, and for the further study of environmental reservoirs of *S. enterica.*

## Introduction

Bacterial genomic epidemiology – the use of genomics-based methods to aid in the epidemiological investigation of communicable diseases – has become an important new tool in the hands of public health laboratories tasked with tracking pathogen outbreaks (Deng et al., 2016; Armstrong et al., 2019). Most outbreaks have been studied retrospectively due to the costs and time involved in analyzing pathogens using pulsed-field gel electrophoresis. However, the introduction of massively parallel sequencing technologies – along with the application of bioinformatics algorithms for assembly, typing, annotation, and phylogenetic analysis – have begun to enable the real-time tracking of outbreaks for infection control and prevention. Whole genome sequencing (WGS) can help public health scientists better understand the origins and dynamics of the outbreak itself (Tang et al., 2017; Armstrong et al., 2019), while providing important information about outbreak strains – such as their serotype, antibiotic susceptibility, potential virulence factors, and toxins – in a single, *in silico* assay (Nadon et al., 2017).

Non-typhoidal *Salmonella enterica* (NTS) are the leading cause of foodborne illness in the United States, and one of the main causes of gastrointestinal disease globally. Worldwide, there are 1.3 billion reported cases of gastroenteritis, 16 million cases of typhoid fever, and 3 million deaths annually attributed to all *S. enterica* infections (Bhunia, 2018), with ca. 535,000 attributed to NTS in 2017 (Parisi et al., 2020). NTS are spread via the fecal-oral route and transmitted via contaminated foods (e.g., poultry, beef, dairy, and eggs), water, and direct animal contact (Silva et al., 2014; Bhunia, 2018). Non-typhoidal *S. enterica* typically causes self-limiting gastroenteritis; however, it can cause invasive disease under certain circumstances (Crump et al., 2015). Effective real-time tracking of new outbreaks requires a large database of *Salmonella* from clinical, food, animal, and environmental sources. As of this writing, over 286,000 draft and complete genomes of *Salmonella* have been sequenced. However, to date only ca. 18,608 are identified as having been isolated from environmental sources such as freshwaters and soils^1^. Irrigation waters are potential sources of *Salmonella* outbreaks in foods (Bell et al., 2015; Liu et al., 2018), and recreational waters may also act as sources for infections in both humans and animals (Levantesi et al., 2012). Unlike other enteric bacteria like *E. coli*, *S. enterica* has high survival rates in aquatic systems and soils, can persist in poultry houses for over a year, and is generally more resistant to environmental fluctuations. It has been postulated that its long-term survival in such secondary habitats facilitates passage to its next host (Winfield and Groisman, 2003). In order to rapidly source human infections in the event of an outbreak, potential environmental reservoirs as well as clinical sources of *S. enterica* need to be monitored.

In keeping with national reform calls in science, technology, engineering, and mathematics (STEM) education to engage all undergraduates in the authentic practice of scientific discovery (National Research Council (US) Committee on Undergraduate Biology Education to Prepare Research Scientists for the 21st Century, 2003; American Association for the Advancement of Science, 2011; National Academies of Sciences, Engineering, and Medicine, 2017) we designed a semester-long course-based undergraduate research experience (CURE) in which upper-division undergraduate students are immersed in the isolation, characterization, and genomic analysis of *S. enterica* isolated from the local environment. As students gain access to more advanced research practices as part of their normal laboratory coursework, a CURE such as this offers students an equitable and inclusive pathway to participate in authentic research (Bangera and Brownell, 2014) and develop skills and knowledge for future careers in the field. This CURE also exposes students to the public health applications of working with foodborne pathogens as well as provides fundamental genomics training applicable to genomic epidemiology (e.g., variant tracking of SARS-COV-2). Students may, as is also described herein, elect to carry out more advanced investigations on their isolates and their genomes. In this paper, we intend to show not only the methods and tools that might be useful for faculty considering implementing a CURE in pathogen genomics, but also a model for how data derived from the isolates and their genomes can be used to address real-world needs and applications in public health genomic epidemiology and in the comparative genomics of foodborne pathogens.

## Materials and Methods

### Course Background and Implementation

The bulk of this research was implemented in a one-semester upper-division laboratory course at James Madison University. The prerequisite for the course is a general microbiology course, both laboratory and lecture. The course consists of two 2 hour-long lab periods per week, as well as regular lab activities outside of the formal lab periods. The course size during this study ranged from 12 to 24 students per semester and has one or two student teaching assistants who have taken the course. Consistent with Auchincloss et al.’s (2014) framework for CURE design, the course employed a collaborative, iterative, discovery-based approach intended to meaningfully engage students in authentic scientific research comparable to that of the community of practice. The course is divided up into a wet lab module, Module 1, and a computational module, Module 2. In Module 1 students work in teams to collect samples, from stream sediments and from poultry litter; then they enrich, purify, identify, and characterize *S. enterica* from these samples. In Module 2 the teams assess read quality, assemble, serotype, and annotate their isolates’ genomes, identify mobile genetic elements, resistance, and virulence genes, etc.

Either module can be implemented independently or they can be employed sequentially over the course of a semester as is done in this course. Details concerning course implementation, including lesson plans for each module, recommended time lines, assessments, etc. are available in Jurgensen et al. (in press). Complete and detailed wet lab and computational protocols, designed and formatted for use in the course, are freely available on the course Open Science Framework (OSF) page^2^. For Module 1, in addition to stream and manure sources of *Salmonella* as described here, *S. enterica* can be isolated from captive or wild reptiles (Marin et al., 2020), amphibians (Ribas and Poonlaphdecha, 2017) and rodents (Meerburg and Kijlstra, 2007; Swanson et al., 2007). Most of the protocols and methods can be modified for use with *E. coli* as well, which can be readily isolated from many urban and rural surface waters. All the work can be done in a typical college microbiology laboratory, albeit following Biosafety Level 2 protocols (see safety documents on OSF^2^). No specialized equipment beyond that found in a typical teaching microbiology laboratory is required, other than perhaps a thermal cycler. Essentially all the work described was carried out by undergraduates each semester over the span of three iterations of the one-semester course, except the nanopore sequencing, phylogenetic analyses, and the advanced aspects of plasmid identification and annotation *–* which were done by undergraduates and an M.S. student in the research lab *–* and the Illumina sequencing, which was done by the Virginia D.C.L.S.

### Environmental Sample Collection

Stream sediment was collected from seven sites on four streams near James Madison University in the Shenandoah Valley of Virginia. Water temperature, salinity, and conductivity were collected using a Sonde^TM^ probe (YSI Incorporated, OH, United States). Metadata was recorded using the mobile application Epicollect5^3^. Stream sediment was collected by inverting a sterile 50 mL Falcon^®^ tube and inserting it straight down into the sediment with a gloved hand while avoiding plant matter and gravel. Each tube was filled approximately 3/4 full with sediment and water. Sediment samples were stored at 4°C during transport and in the lab until processing. Poultry litter was acquired from a chicken farm in northern Rockingham County, Virginia, housing approximately 150,000 birds. The farmer was provided with a clean plastic container for filling with litter. Litter was stored at room temperature (20–25°C) until processing.

### Isolation of *Salmonella enterica* From Stream Sediment/Poultry Litter

**FIGURE 1:**
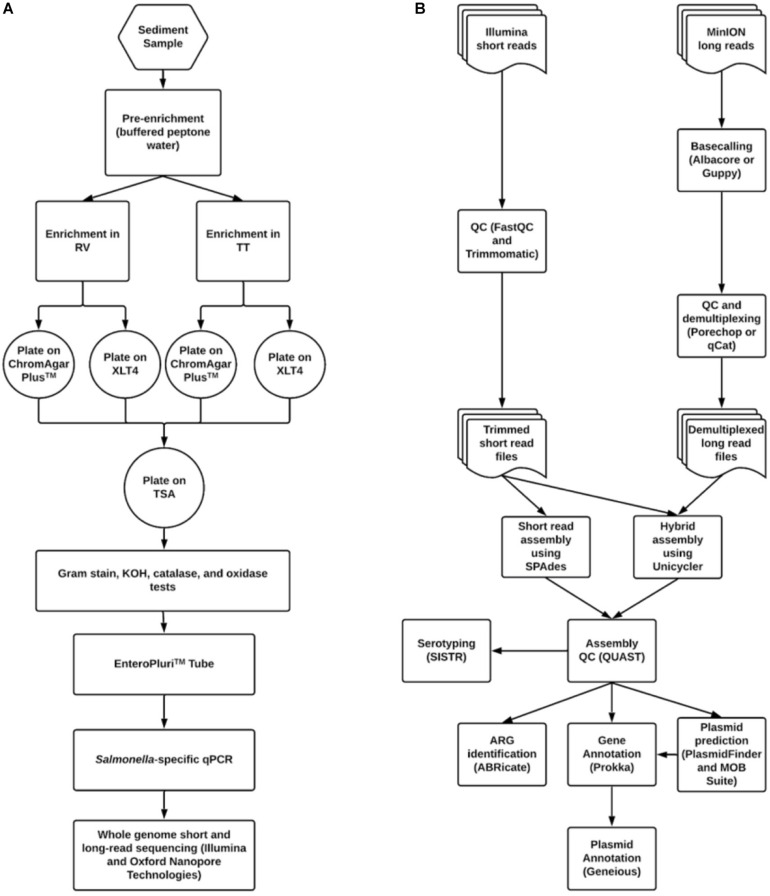
Workflows for **(A)** *Salmonella* isolation and **(B)** bioinformatic data processing and analysis.

An outline of the methods used to isolate and identify *S. enterica* in sediment and poultry samples is shown in Figure 1A. The procedure was based loosely on the United States FDA Bacteriological Analytical Manual *Salmonella* isolation protocol (Andrews et al., 2014). Both sediment and litter were processed in the same manner. Pre-enrichment began within 24 h of sample collection. Fifty grams of sediment or litter were transferred to sterile 250 mL Erlenmeyer flasks in duplicate. One hundred milliliters of buffered peptone water (10 g peptone, 5 g NaCl, 7 g Na_2_HPO_4_, 3 g KH_2_PO_4_ per liter) were added to each flask and mixed by swirling. Pre-enrichments were incubated with shaking at 35°C for 16–22 h. After incubation, 1 mL of supernatant was added to screw-cap tubes containing 10 mL of either Tetrathionate (TT) or Rappaport-Vasilliadis (RV) broth. TT was made in one liter batches (5 g polypeptone, 1 g bile salts, 10 g CaCO_3_, 30 g Na_2_S_2_O_3_ per liter) with additional 20 mL iodine-potassium iodide (5 g KI, 6 g iodine resublimated) added. RV was also made in one liter batches consisting of 100 mL magnesium chloride solution (400 g MgCl_2_ 6H_2_O per liter) and 10 mL malachite green oxalate solution (0.4 g malachite green oxalate per liter) to one liter of broth base (5 g tryptone, 8 g NaCl, 1.6 g KH_2_PO_4_ per liter). RV was autoclaved prior to the addition of pre-enrichment. All enrichments were shaken at 42°C for 5 days.

One hundred microliter aliquots from each enrichment were spread-plated onto Xylose Lysine Tergitol-4 (XLT4) agar (Becton Dickinson, Franklin, NJ, United States) and CHROMagar^TM^ *Salmonella* agar (DRG International Inc., Springfield, NJ). Plates were incubated at 35°C for 16–22 h. Putative *Salmonella* colonies on each medium were identified based on morphology and then streaked onto the complementary agar. If *Salmonella*-like morphology was seen on both media, then colonies were streaked onto tryptic soy agar (TSA) (Becton Dickinson, Franklin, NJ, United States) for purification.

### Identification of *S. enterica*

Gram-negative, oxidase-negative, catalase- and KOH-positive isolates were grown in an EnteroPluri^TM^ tube (Becton-Dickinson, Franklin Lakes, NJ, United States). The EnteroPluri^TM^ tube allows for the simultaneous inoculation of multiple media types and the execution of 15 separate biological reactions. Isolates identified as *Salmonella* were then subjected to an endpoint PCR using primers targeting the *Salmonella*-specific gene *invA (Malorny et al., 2003)* for confirmation. Briefly, a small number of cells were acquired by touching an inoculating needle to a colony. The cells were added to 5 μL of ddH_2_O in a 0.2 mL PCR tube and lysed in a thermocycler at 95°C for 5 minutes. A master mix was made consisting of 12.5 μL of 2X AmpliTaq Gold^®^ (ThermoFisher Scientific, Waltham, MA, United States) (0.625 U AmpliTaq Gold DNA polymerase, 30 mM Tris/HCl, pH 8.05, 100 mM KCl, 400 μM each dNTP, 5 mM MgCl_2_), one μL of both *invA* 139 primer (5′-GTGAAATTATCGCCACGTTCGGGCAA-3′) and *invA* 141 primer (5′-TCATCGCACCGTCAAAGGAACC-3′) at 10 μM concentrations, and 5 μL of ddH_2_O. Twenty microliters of master mix were added to the lysed cell mixture and run according to the program of Malorny et al. (2003): 95°C for one minute followed by 36 cycles of 95°C for 30 s, 64°C for 30 s, and 72°C for 30 s, then a final extension at 72°C for four minutes. For agarose gel electrophoresis, eight microliters of PCR product were added to 2 μL of 5X loading dye prior to loading. The gel was run at 5 V/cm for ca. 120 min and stained with 0.5% GelRed (Biotium Inc., Fremont, CA, United States) for 20-30 min followed by de-staining with ddH_2_O for 5 min. Bands were visualized using a UV transilluminator. A band size of 285 bp was expected for an *invA* positive result. For long term storage, one mL of culture was combined with one mL of sterile glycerol in a 2 mL cryogenic freezer tube and stored at −80°C. Strain names were derived from the initials of student teams that isolated them.

### Genomic DNA Extraction for Oxford Nanopore DNA Sequencing

Cells were grown in tryptic soy broth for 16–20 h. The Qiagen^TM^ DNeasy Blood and Tissue Kit (Qiagen, Germantown, MD, United States) was used following the manufacturer’s instructions for Gram-negative bacteria with some modifications: cell density was not assayed prior to extraction, during the incubation step with proteinase K the length of incubation was kept to a maximum of 1 h, and after elution with ddH_2_O, the DNA was left to dissolve for 24 h at 4°C.

### Genomic DNA Sequencing

Short read sequence data were generated by the United States FDA Center for Food Safety and Applied Nutrition, as well as the Virginia State Department of Consolidated Laboratory Services. Both carried out sequencing on an Illumina^®^ MiSeq sequencer using either a 300 cycle (2 × 151) or 500 cycle kit (2 × 251). Raw sequence data were uploaded to Illumina BaseSpace, GalaxyTrakr, and to NCBI’s Sequence Read Archive (SRA).

For nanopore sequencing, DNA quality and concentrations were assessed using a Synergy H1 Multi-Mode Reader (BioTek Instruments, VT, United States) and a Qubit 2.0 fluorometer, respectively. An OD_260/280_ of 1.8–2.0 was used as the quality cutoff. DNA concentration was determined using the Qubit dsDNA broad range kit. DNA samples were concentrated for samples where when necessary using Microcon^®^ centrifugal filters (Merck Millipore Ltd., MA, United States) according to the manufacturer’s instructions.

Prior to nanopore sequencing, a flow cell QC was performed according to the manufacturer’s instructions. Library preparation was done using the rapid barcoding kit (SQK-RBK004) according to the manufacturer’s instructions (version RNK_9054_v2_revA_23Jan2018; Oxford Nanopore Technologies, Oxford, United Kingdom). Sequencing on the ONT MinION proceeded for up to 48 h using a FLO-MIN106 flowcell (R9.4.1 pore type).

### Sequence Data Quality Control and Analysis

Sequence data were processed according to the pipeline shown in Figure 1B. Bioinformatic processing and analyses were done in GalaxyTrakr (Gangiredla et al., 2021) or using the command line interface on a computer with an Ubuntu 16.04 LTS operating system. Short read data were quality checked using FastQC^4^ version 0.72 or 0.69. Low-quality data was removed using Trimmomatic version 0.36.4 or 0.36.3 (Bolger et al., 2014). Trimmomatic operations consisted of sliding window trimming using a window of four bases with an average quality cutoff of 20, then an overall average quality trimming with a cutoff of 27, and finally a minimum length trimming with a cutoff of 70% the maximum read length (i.e., for data with read length of 251, reads below 75 bp were removed). Trimmed reads were again run through FastQC. Assembly using short read data was carried out using SPAdes version 3.11.1 with default options and specified k-mer values of 21, 33, 55, 77, 99, and 127 (Bankevich et al., 2012).

For long read data, basecalling was performed using either Albacore version 2.2.7 or Guppy 3.0.3 (Oxford Nanopore, Oxford, United Kingdom). Adapter removal and demultiplexing was performed using either Porechop^5^ version 0.2.3 or Qcat^6^ version 1.0.6. These data were used in conjunction with short read data to carry out a hybrid assembly using Unicycler version 0.4.1.1 (Wick et al., 2017). Assembly quality was assessed using QUAST version 4.6.3 (Gurevich et al., 2013). Assembly quality thresholds used were: N50 > 200,000, number of contigs <200, sequence length ca. 4.4 to 5 Mbp.

### Serotyping of *Salmonella* Isolates

Serotyping was done *in silico* using Seqsero2 version 2.0 and SISTR version 1.0.2 on GalaxyTrakr. Additionally, serotyping using SMART PCR (Leader et al., 2009) was carried out on isolates HJ-01 to HJ-26. Trimmed short reads (as FASTQ files) were used as inputs to Seqsero2. Assembled genomes (either short read only or hybrid assemblies, as FASTA files) were used as inputs to SISTR. Agreement between the two *in silico* tools and, if necessary, SMART PCR were used to determine the consensus serotype of a given isolate.

### Antimicrobial Resistance Genotyping

Antimicrobial resistance genes (ARGs) were identified using ABRicate^7^ versions 0.7.0, 0.8.0, 0.8.7, and ResFinder version 3.2 (Zankari et al., 2012). ABRicate was used through GalaxyTrakr and the command line. Default settings were used on both platforms with two exceptions: a minimum identity cutoff of 80% was specified and the database used was NCBI. ResFinder was run through the Center for Genomic Epidemiology website^8^. For ResFinder, the “acquired ARGs” option was used and final assemblies were submitted.

### Phylogenetic Analysis

Eighty-eight *S. enterica* isolates were used to generate a phylogenetic tree in Enterobase (Zhou et al., 2020). A neighbor-joining tree was generated using the algorithm RapidNJ in Enterobase from Enterobase’s cgMLST scheme, a set of alleles for 3,002 loci that make up *S. enterica’s* core genome. GrapeTree (Zhou et al., 2018) and TreeGraph2 (Stöver and Müller, 2010) were used to visualize the tree. The tree was rooted using the genome of *S. enterica* subspecies *salamae* strain 1315 K. Phandango (Hadfield et al., 2018) was used to visualize source and serotype metadata mapped onto the tree.

### *In silico* Identification and Annotation of Plasmids

MOB_Suite version 1.4.8 (Robertson and Nash, 2018) and PlasmidFinder’s most recent version (Carattoli et al., 2014) were used to identify potential plasmids from short read-only assemblies. PlasmidFinder was used through the Center for Genomic Epidemiology website. The Enterobacteriaceae database was employed, with an identity cutoff of 90% and a minimum coverage cutoff of 80%. For MOB_Suite, the mob_recon command’s basic options, which require only an input FASTA file and an output directory location, were used, along with the mob_typer command for plasmid typing. MOB_Suite is now available on GalaxyTrakr^9^.

Annotation of plasmids was done using the commercial platform Geneious Prime^TM^ 2019 (Biomatters Ltd., San Diego, CA, United States). Bandage (Wick et al., 2015) was used to visualize assemblies and to identify potential plasmids from hybrid assembly graphs generated by Unicycler. Plasmid sequences were downloaded as FASTA files from Bandage graphs and submitted to Prokka version 0.13.0 (Seemann, 2014) for automatic annotation using the default parameters: Locus tag prefix PROKKA, Locus tag counter increment = 1, GFF version = 3, no forced GenBank/ENA/DDJB compliance, minimum contig size = 200, kingdom = Bacteria, genetic code = 11, similarity e-value cut-off = 0.000001. Not used: “gene” feature for “CDS” feature, genus-specific BLAST database, improve gene prediction for highly fragmented genomes, fast mode, searching for ncRNAs, rRNA search with Barnap, and tRNA search with Aragorn. In addition, identification of unidentified coding sequences was attempted using BLASTx. Parameters used for BLASTx were: max target sequences = 100, expect threshold = 10, word size = 6, and maximum number of matches in a query = 0. The BLOSUM62 matrix was used for scoring. GFF3 files from Prokka were uploaded to Geneious for plasmid mapping and editing.

## Results

### Course Development

Due to the large amount of poultry and cattle farming in the central Shenandoah Valley of Virginia, we hypothesized that *Salmonella* could be isolated from area streams that drain these farms. If this proved to be true, we intended to develop an elective upper-level research course in which undergraduates would learn to isolate and identify *Salmonella* and to assemble and annotate their genomes. We hoped thereby also to initiate a long-term study on *Salmonella* in streams in our area and to develop the methods necessary for other colleges and universities to replicate the course or a portion thereof. Our focus was particularly on the sediments of streams, as these have been shown to harbor a more stable population of introduced bacteria, particularly *Enterobacteriaceae*, than does the water column itself (Hendricks, 1971; Burton et al., 1987; Pachepsky and Shelton, 2011). Initial attempts to isolate *Salmonella* from sediments in various agriculturally impacted streams were unsuccessful. However, after lengthening the time of enrichment from 24 h to 5 days (Figure 1A) we were able to routinely isolate *S. enterica* from all of our tested stream sites.

We wished to use WGS of these isolates both to understand the population-level dynamics of these *Salmonella* and to train students in some basic bioinformatics methods in microbial genomics. A bioinformatics workflow was developed and used for the QC, assembly, and annotation of *Salmonella* genomes (Figure 1B). This workflow was carried out primarily using GalaxyTrakr, an FDA Galaxy instance developed particularly for the use of public health laboratories for analyzing the genomes of foodborne pathogens (Gangiredla et al., 2021).

These isolation and computational protocols were piloted in a new course, BIO346 *Bacterial Discovery*, beginning in the spring of 2018. Over three separate semesters, CURE students (*n* = 52) isolated and characterized 15 *S. enterica* strains. Three of these students went on to isolate an additional 34 strains in the Herrick research lab. These combined with the 39 strains isolated during the methods development for the course resulted in a total of 88 strains isolated from October 2016 through September 2018 (Table 1). Of these, 83 strains were isolated from the sediment of seven sites on four streams and five strains from a broiler poultry house, all in the Shenandoah River watershed Rockingham County, Virginia.

**TABLE 1:** *Salmonella enterica* strains isolated from stream sediment and poultry litter in the Shenandoah Valley of Virginia from October 2016 through September 2018.

**Isolate**	**SRR number**	**Collection date**	**Source^*a*^**	**Source type**	**Serotype**	**MLST^*b*^**	**cgMLST^*c*^**
HJ-01	SRR5886281	2016/10/02	MC	Sediment	Give	654	97788
HJ-02	SRR5886286	2016/10/02	MC	Sediment	Give	654	80760
HJ-03	SRR5886299	2016/10/02	PR	Sediment	Uganda	684	80845
HJ-04	SRR5886298	2016/10/02	PR	Sediment	Uganda	684	70491
HJ-05	SRR5886283	2016/10/16	CC11	Sediment	Litchfield	214	80915
HJ-06	SRR5886290	2016/10/16	CC11	Sediment	Schwarzengrund	96	80849
HJ-07	SRR5886279	2016/10/16	PR	Sediment	Muenster	321	80796
HJ-08	SRR5886351	2016/10/16	PR	Sediment	Muenster	321	80796
HJ-09	SRR5886350	2016/10/16	CC704	Sediment	Mbandaka	413	80794
HJ-10	SRR5884063	2016/12/05	MC	Sediment	Anatum	64	80537
HJ-11	SRR5884068	2016/12/05	CC704	Sediment	Schwarzengrund	96	80539
HJ-12	SRR5884053	2016/12/05	CC704	Sediment	Senftenberg	14	80536
HJ-13	SRR5884069	2017/01/15	CC11	Sediment	Hadar	33	80533
HJ-14	SRR5884070	2017/01/15	CC11	Sediment	Hadar	33	80527
HJ-15	SRR5884058	2017/01/15	CC11	Sediment	Hadar	33	80538
HJ-16	SRR5884066	2017/02/01	L	Litter	Cerro	367	80542
HJ-17	SRR5884067	2017/02/01	L	Litter	Typhimurium	19	80529
HJ-18	SRR5884056	2017/02/01	L	Litter	Typhimurium	19	80535
HJ-19	SRR5884062	2017/02/01	L	Litter	Typhimurium	19	80530
HJ-20	SRR5884079	2017/02/01	L	Litter	Typhimurium	19	80534
HJ-21	SRR5884080, SRR13268785^d^	2017/02/05	CC11	Sediment	Typhimurium	19	80528
HJ-22	SRR5884077	2017/02/26	CC704	Sediment	Muenchen	112	80532
HJ-23	SRR5884081	2017/02/26	CC704	Sediment	Muenchen	112	80526
HJ-24	SRR6832866, SRR13268782^d^	2017/09/10	PR	Sediment	Montevideo	138	111799
HJ-25	SRR6832877	2017/09/10	PR	Sediment	Montevideo	138	169196
HJ-26	SRR6832873	2017/09/10	MC	Sediment	Senftenberg	14	111800
HJ-27	SRR6366729	2017/10/22	CC704	Sediment	Cerro	367	101373
HJ-28	SRR6367403	2017/10/22	CC704	Sediment	Cerro	367	101373
HJ-29	SRR6369106, SRR13268783^d^	2017/10/22	CC704	Sediment	Anatum	64	11895
HJ-30	SRR6367413	2017/10/22	CCP	Sediment	Braenderup	22	4601
HJ-31	SRR6367404	2017/10/22	CCP	Sediment	Braenderup	22	101420
HJ-32	SRR6367414	2017/10/22	CCP	Sediment	Braenderup	22	4601
HJ-33	SRR6367467	2017/10/22	MC	Sediment	Montevideo	138	101411
HJ-34	SRR6832876	2018/01/21	CCP	Sediment	Braenderup	22	113399
HJ-35	SRR6832896	2018/01/21	CCP	Sediment	Braenderup	22	4601
HJ-36	SRR6832925	2018/01/21	CCP	Sediment	Typhimurium	19	113353
HJ-37	SRR6832911	2018/01/21	CCP	Sediment	Typhimurium	19	111797
HJ-38	SRR6832904, SRR13268784^d^	2018/01/21	CCP	Sediment	Typhimurium	19	111798
HJ-39	SRR6832916	2018/01/21	CCP	Sediment	Typhimurium	19	111391
FHS-01	SRR6832910	2018/01/23	CC704	Sediment	Montevideo	138	166542
FHS-02	SRR6832913, SRR13268786^d^	2018/01/23	CC704	Sediment	Infantis	32	111896
FHS-04	SRR6832912	2018/01/23	CC704	Sediment	Montevideo	138	152383
DG-01	SRR8360264	2018/05/20	PR	Sediment	Muenchen	112	131452
DG-02	SRR8360271	2018/05/20	PR	Sediment	Montevideo	138	131598
DG-03	SRR8104581	2018/05/20	PR	Sediment	Montevideo	138	131599
DG-04	SRR8104579	2018/05/20	CC11	Sediment	Hadar	33	131627
DG-05	SRR8104582	2018/05/20	CC11	Sediment	Hadar	33	131627
DG-06	SRR7504390	2018/05/20	CC11	Sediment	Hadar	33	131627
DG-07	SRR7506701	2018/05/20	CC11	Sediment	Hadar	33	136579
DG-08	SRR7506695	2018/05/20	CC704	Sediment	Typhimurium	19	136587
DG-09	SRR7506699	2018/05/20	CC704	Sediment	Typhimurium	19	131813
DG-10	SRR8360260	2018/05/20	CC704	Sediment	Typhimurium	19	153131
DG-11	SRR7504359	2018/05/20	CC704	Sediment	Typhimurium	19	153155
DG-12	SRR7506710	2018/05/20	CC704	Sediment	Typhimurium	19	153158
DG-13	SRR7499244	2018/05/20	CC704	Sediment	Typhimurium	19	153152
DG-14	SRR7499253	2018/05/20	MC	Sediment	Meleagridis	463	135814
DG-15	SRR7499245	2018/05/20	MC	Sediment	Meleagridis	463	135813
DG-16	SRR7499272	2018/05/20	MC	Sediment	Cerro	367	135849
DG-17	SRR7499280	2018/05/20	MC	Sediment	Meleagridis	463	135804
DG-18	SRR7499278	2018/05/20	MC	Sediment	Meleagridis	463	135809
DG-19	SRR7889322	2018/05/20	CC11	Sediment	Schwarzengrund	96	144617
DG-20	SRR7878396	2018/05/20	CC704	Sediment	Alachua	1298	144057
DG-21	SRR7889352	2018/05/20	CCP	Sediment	Mbandaka	413	144638
DG-22	SRR7878395	2018/05/20	BR	Sediment	Mbandaka	413	144059
AP-01	SRR8179982	2018/09/03	CC11	Sediment	Bareilly	2553	153210
AP-02	SRR8179943	2018/09/03	CC11	Sediment	Bareilly	2553	153210
AP-03	SRR8179958	2018/09/03	CC11	Sediment	Bareilly	2553	153210
AP-04	SRR8179911	2018/09/03	CC11	Sediment	Bareilly	2553	153210
BES-01	SRR8179966	2018/09/03	CC704	Sediment	Reading	412	153206
BES-02	SRR8179907	2018/09/03	CC11	Sediment	Reading	412	153208
PPL-01	SRR8179980	2018/09/03	CC11	Sediment	Braenderup	22	4601
PPL-02	SRR8179892	2018/09/03	CC11	Sediment	Braenderup	22	4601
WEK-01	SRR8179901	2018/09/03	CC704	Sediment	Montevideo	138	153130
WEK-02	SRR8179936	2018/09/03	CC11	Sediment	Mbandaka	413	153196
WEK-03	SRR8179927	2018/09/03	CC11	Sediment	Braenderup	22	4601
WEK-04	SRR8179981	2018/09/03	CC11	Sediment	Mbandaka	413	153188
WMD-01	SRR8570270	2018/09/30	PR	Sediment	Muenchen	112	164635
WMD-02	SRR8570265	2018/09/30	CC11	Sediment	Give	654	169281
WMD-03	SRR8570271	2018/09/30	MC	Sediment	Montevideo	138	164641
WMD-04	SRR8570267	2018/09/30	MC	Sediment	Montevideo	138	164640
WMD-05	SRR8570264	2018/09/30	CCA	Sediment	Muenster	321	164639
WMD-07	SRR8573695	2018/09/30	PR	Sediment	Montevideo	138	164637
WMD-08	SRR8570269	2018/09/30	CCA	Sediment	Montevideo	138	164636
WMD-09	SRR8570273	2018/09/30	MC	Sediment	Montevideo	138	164643
WMD-10	SRR8570263	2018/09/30	CCA	Sediment	Cerro	367	164638
WMD-11	SRR8570268	2018/09/30	MC	Sediment	Typhimurium	19	164642
WMD-12	SRR8570266	2018/09/30	BR	Sediment	Give	654	164644
WMD-13	SRR8570272	2018/09/30	CCA	Sediment	Cerro	367	164645

*^a^PR: Pleasant Run, CC11: Cook’s Creek Rt. 11, CC704: Cook’s Creek Rt. 704, CCP: Cook’s Creek Park, CCA: Cook’s Creek Arboretum, MC: Muddy Creek, BR: Black’s Run, and L: poultry litter.*

*^b^MLST: 7-gene multilocus sequence type.*

*^c^cgMLST: core-genome multilocus sequence type, Enterobase scheme (Zhou et al., 2018).*

*^d^Oxford Nanopore fastq SRR numbers.*

### Distribution of Sero- and Genotypes

**FIGURE 2:**
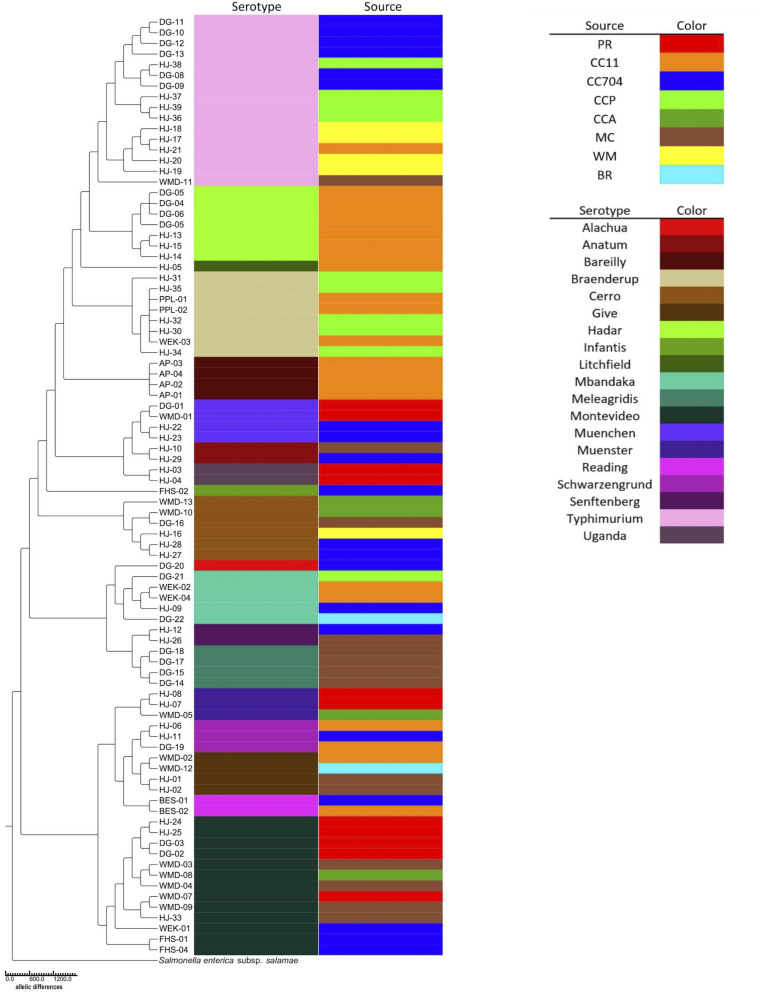
Neighbor-joining cgMLST phylogenetic tree of 88 *S. enterica* isolates generated using the Enterobase GrapeTree RapidNJ algorithm. Branch lengths correspond to the number of allelic differences between isolates. The tree was rooted using *S. enterica* subspecies *salamae* strain 1315K. Isolate source and serotype are indicated by colored bars.

Serotypes were determined by consensus of both SeqSero2 and SISTR and, for strains HJ1 to HJ26, using SMART PCR (Leader et al., 2009). SeqSero2 was unable to serotype strains HJ-02, HJ-04, HJ-13, and HJ-20; however, SMART PCR agreed with SISTR in its serotype determination for these isolates. The 88 isolates were distributed in 19 serotypes, of which the largest number (*n* = 16) were Typhimurium. The serotypes exactly aligned with the seven-gene multilocus sequence type (MLST), i.e., each serotype corresponded with exactly one MLST. It was found that not only were different serotypes isolated from within the same source, but the same serotype was isolated from different sources as well (Figure 2). Also, while certain serotypes were confined to a single source (e.g., Hadar, Bareilly, and Meleagridis), others such as Typhimurium, Montevideo, and Cerro were isolated from a number of different sources.

Among the 88 isolates, 75 distinct core-genome multilocus sequence types (cgMLST) were found (Table 1). Thirteen were apparently duplicates, having the same cgMLST as at least one other strain isolated from the same source on the same date. However, six serotype Braenderup strains with the same cgMLST (#4601; strains HJ-30, -32, and-35, PPL-01 and -02, and WEK-03) were isolated from different areas of Cooks Creek over the course of three samplings in 1 year. They were isolated in October 2017 (HJ-30 and HJ-32) and January 2018 (HJ-35) from the upstream CCP site, and in September 2018 the same sequence type was isolated three times (PPL-01, PPL-2, and WEK-03) downstream at the CC11 sampling site (Table 1).

### Antibiotic Resistance and Plasmid Characterization

**FIGURE 3:**
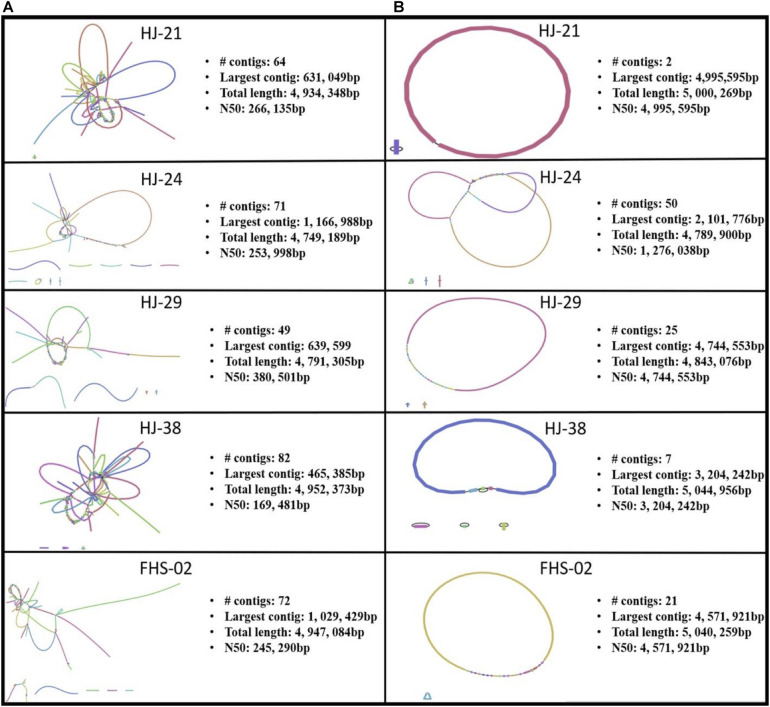
Bandage visualizations of five *Salmonella enterica* plasmid assemblies using **(A)** short read sequence data only, and **(B)** hybrid assemblies using short and long read data.

Of the 88 isolates collected, 31 were found to contain one or more ARGs (data not shown). Of the 31 isolates with ARGs, 26 were predicted using MOB_Suite and/or PlasmidFinder to house one or more plasmids (data not shown). However, both these tools use assemblies based on short read data as inputs. Short read sequencing data, though highly accurate, result in fragmented, discontiguous assemblies (Figure 3A). A hybrid, whole genome assembly approach, incorporating both short and long read data, allowed for the resolving of complete or nearly complete genomes (Figure 3B).

**FIGURE 4:**
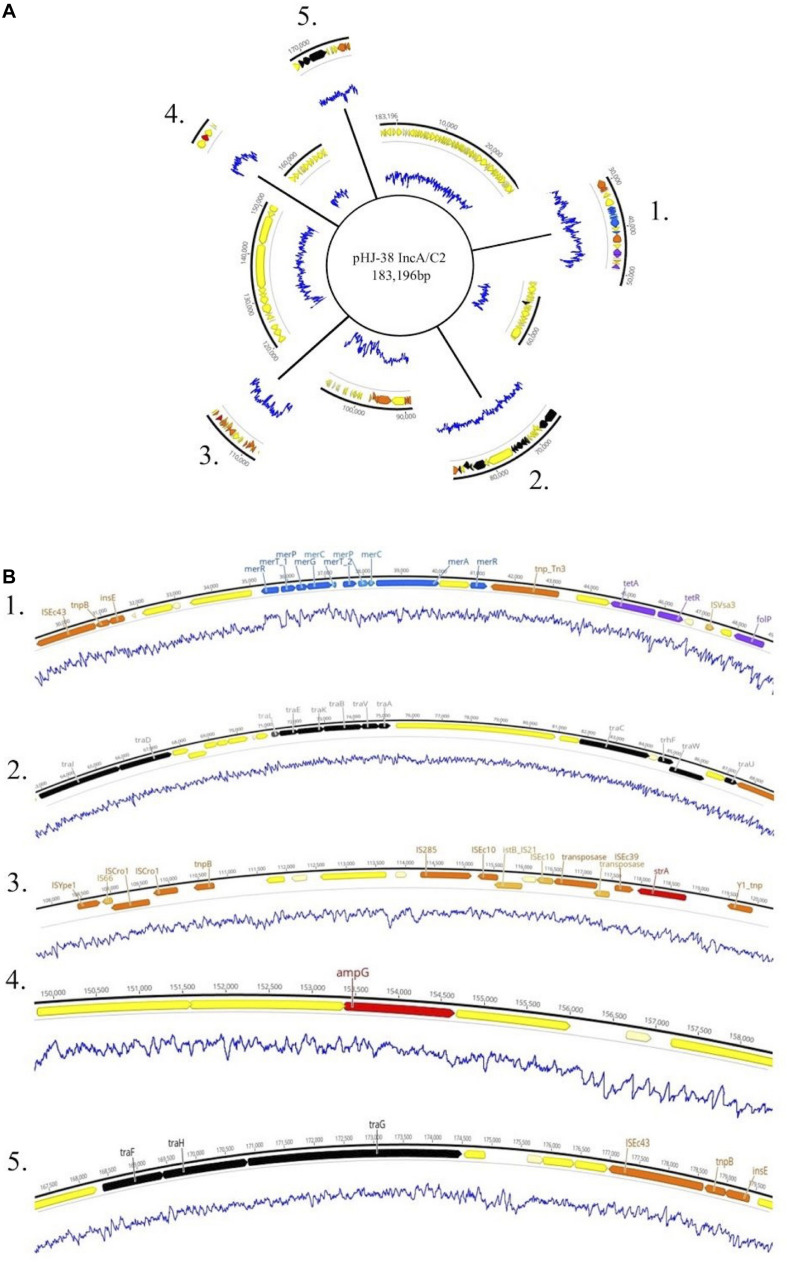
**(A)** Genetic map of the 184 kb IncA/C2 plasmid pHJ-38. Excised regions 1–5 of the plasmid contain mobility associated genes and genetic elements, antimicrobial and metal resistance genes, virulence genes, and other regions of interest. **(B)** Annotated gene regions containing heavy metal resistance genes (blue); virulence factors (light green); colicin genes (dark green); toxin-antitoxin gene cassettes (pink); genes associated with transposons, IS elements, or integrons (orange); and conjugation genes (black) are shown. Antimicrobial resistance genes identified by both Prokka and Abricate (purple) or by Prokka alone (red) are also shown. Yellow and cream-colored gene regions indicate hypothetical proteins. Blue lines indicate percent GC content.

**FIGURE 5:**
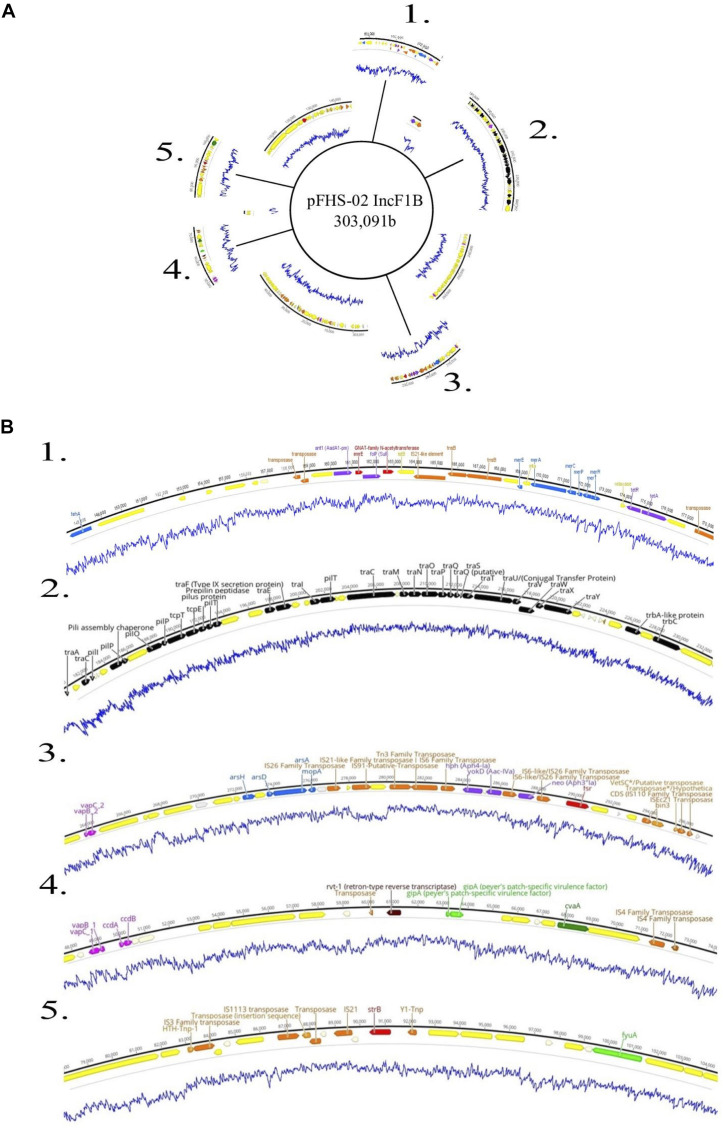
**(A)** Genetic map of the 303 kb IncF1B plasmid pFHS-02. Regions 1–5 of the plasmid containing mobility associated genes and genetic elements, antimicrobial and metal resistance genes, virulence genes, and other regions of interest are shown excised. **(B)** Annotated gene regions; genes and labels are colored as indicated in Figure 4. Blue lines indicate percent GC content.

Plasmids identified in isolates that contained one or more ARGs were annotated using Prokka (Seemann, 2014). Two of these plasmids are shown in Figures 4, 5. Plasmid pHJ-38 was an IncA/C2 plasmid found to have multiple ARGs as predicted by ABRicate and ResFinder (Figure 4). Other notable features identified were multiple heavy metal resistance genes and *tra* genes. *Tra* genes are essential for plasmid conjugation between potential host bacteria (Zatyka and Thomas, 1998; Thomas, 2000) and suggest this is a self-transmissible plasmid. Similarly, in the IncF1B plasmid pFHS-02, multiple antibiotic and heavy metal resistance genes were identified (Figure 5). Along with *tra* genes, *pil* genes were also present in both plasmids. These genes encode a different pilus than those typically encoded by *tra* genes, one usually associated with conjugation in liquid environments (Bradley, 1984; Zatyka and Thomas, 1998). Plasmid pFHS-02 was also found to contain multiple toxin-antitoxin (or “plasmid addiction”) systems. These systems exist to ensure a plasmid’s successful replication during host cell division (Hayes, 2003).

### Assessment of Educational Outcomes

To determine the impact of the CURE on the development of participants’ content knowledge, skills, and attitudes in the domain, direct measures of classroom performance (see Jurgensen et al., in press for an extended discussion of the employed assessments) were partnered with indirect self-report data collected via in-class surveys near the beginning and end of the term. During the first 2 weeks of class, students were asked to complete a short set of closed-response items regarding their academic and demographic background with a limited number of open-ended questions focused on their course expectations. At the end of the term, students responded to Likert scale questions drawn from multiple validated instruments (Hurtado and Carter, 1997; Smith et al., 2013; Corwin et al., 2015b; Hanauer et al., 2017; Maltese et al., 2017) combined with closed-response and open-response questions designed for this study to capture data pertaining to changes in academic/career interests and course design. Survey data were collected online and aggregately analyzed from students in three consecutive semesters of the course (fall 2018, spring 2019, fall 2019) with approval by the university’s Institutional Review Board (IRB). Student participants (*n* = 50) were all biology majors with 74% self-identifying as female, 72% white, 30% first-generation students, 8% had participated in an apprenticeship-like undergraduate research experience, and 26% were working >15 h per week “to make ends meet,” which suggests the course offered an opportunity to engage students who may not be able to volunteer as a traditional, mentored undergraduate researcher due to financial considerations (Bangera and Brownell, 2014). Here, given the focus of this special issue, we report on a subset of post-survey items used to address questions as to how participation in the research course influenced students’ academic/career interest and persistence in science, while a description of conferred cognitive outcomes and perceptions of the course is presented in Jurgensen et al. (in press).

#### Measures

Multiple data points were collected to gain an understanding of how the experience influenced students’ academic and career interests. First, using an approach comparable to Maltese and Harsh (2015), students were asked at the onset and end of term to report their intentions (e.g., STEM graduate school, professional school, non-STEM career, and unsure) upon graduation. Exiting students were also prompted if their future intentions had shifted over the term, and if so, they were asked to qualitatively describe whether the course had influenced their plans. Additionally, we also asked students to rate their level of interest in research and science, in general, after the class on a five-point scale. Then, students completed eight parallel items using five-point Likert-type scales (1 = far less interested, 5 = far more interested) created for this project that assessed their interest in pursuing future coursework and research opportunities in specific course topics (i.e., microbiology, molecular biology, bioinformatics, genomics, and microbial ecology) via the stem prompt, “After this class, I am [interest level] in future [coursework or research opportunities] in [topic].” Finally, we used the Persistence in the Sciences survey (PITS) and measures of sense of belonging to assess conferred psychosocial outcomes to research course participants that are relevant to STEM persistence (Chemers et al., 2011; Estrada et al., 2011). The PITS instrument (Hanauer et al., 2016) is designed to measure psychological variables related to student persistence (e.g., project-ownership, self-efficacy, science identity), and has been previously used to assess the effectiveness of CURE educational designs (Hanauer et al., 2017; Corwin et al., 2018; Cooper et al., 2019; Zelaya et al., 2020). Each item is scored on a scale ranging from one to four or five (depending on variable), and then averaged to form a composite variable score. In addition, we also used items modified from Hurtado and Carter (1997) and Smith et al. (2013) to measure sense of belonging assessed using five-point Likert-type scales (1 = strongly disagree, 5 = highly agree).

#### Findings

Students entered the course largely with the intentions of attending graduate school in a STEM field (42%) or professional school (42%) after degree completion, with fewer intending to seek immediate employment in STEM fields (12%) or who were unsure of their respective plans (4%). Fifty-six percent of participants reported their academic or career plans changed to some degree during the term, with 36% of all students specifically identifying that the course influenced their future intentions. Most often, similar to interest shifts observed in mentored undergraduate research experience (Maltese and Harsh, 2015), students indicated that the experience refined or triggered their interest through exposure to fields of study and careers related to genomic epidemiology. For instance, two students stated in open-ended questions that “*[The course] opened the door and exposed me to genomics and bioinformatics in which I had never thought about in my future career*” and “*It significantly influenced my future plans and [sic] considering to attend a graduate school specifically [to study] foodborne pathogens in relation to the environment so that I possibly could work for the CDC or FDA in the future.*” A notable proportion of students upon exiting the course indicated that the experience enhanced their interest, to some degree, in coursework in bioinformatics (40%), microbial ecology (33%), molecular biology (24%), and genomics (22%). While the balance of respondents largely reported no change in their prior interests, a small subset (<10%) indicated decreased interests in these topical areas resultant of class participation. A modest shift in interest in microbiology coursework was also noted (8% increase, 92% no change), which likely reflects the preexisting interests of students that opted into such an upper-level course. Results also showed that 77% percent of students identified that course participation increased their overall interest in pursuing future research opportunities. More specifically, a fair proportion of students indicated that the experience enhanced their interests, to some degree, in research in bioinformatics (42% of respondents), molecular biology (34%), genomics (31%), and microbial ecology (24%) research with a smaller shift for microbiology (16%). On the other hand, approximately 20% of students indicated that they became less interested in research over the term in the respective areas of microbial ecology, bioinformatics, and genomics. Together, these results suggest that the research course refined student interest by the opportunity to test the proverbial waters of different fields through authentic practice, which may guide their later academic and career intentions in a means comparable to that of a traditional undergraduate research experience (Hunter et al., 2007; Maltese and Harsh, 2015).

In addition to observing shifts in academic and/or career interests, we sought to assess the impact of course participation on psychosocial aspects often correlated with academic success and STEM persistence (Perez et al., 2014; Trujillo and Tanner, 2014). Most students (80, 72, and 76%, respectively) reported on the post-survey that participation in the course directly contributed to a greater sense of belonging to the department, campus community, and scientific community. Students reported high ratings on PITS items measuring their science-identity (*M* = 4.52, SD = 0.46), self-efficacy (*M* = 4.32, SD = 0.77), cognitive project ownership (*M* = 4.22, SD = 0.59), and networking (*M* = 4.10, SD = 0.71) as related to course participation. These ratings are comparable to or higher than those reported in previously published studies on biologically focused CUREs (Corwin et al., 2015a; Hanauer et al., 2017; Cooper et al., 2019; Zelaya et al., 2020) that use the same instruments (Hanauer and Dolan, 2014; Hanauer et al., 2016) to assess student outcomes. The overall pattern of early findings suggests the research course contributes to psychosocial outcomes that influence STEM persistence, though additional data is needed to allow comparisons to be drawn between groups to assess the impact of participation of the experience on *all* students.

## Discussion

Animal husbandry plays a prominent role in the economy of the Shenandoah Valley of Virginia, with an estimated 159 million chickens, 16 million turkeys, and over a half million cattle raised in 2012 in four counties alone. These were estimated to produce over 400 tons and 1.28 billion gallons of manure in that year^10^. We hypothesized that agricultural runoff, particularly from poultry and cattle, would result in detectable enteric pathogens such as *Salmonella* in these streams and rivers. In the approximately 15 months before beginning this course, and then over three semesters of the course itself, the sediments of seven sites on four streams and one poultry house were sampled and a total of 88 distinct *S. enterica* strains were isolated. Standard microbiological techniques along with the Enteropluri tube and PCR of the *invA* gene were used to verify the isolates’ identity (Figure 1A). Students typically characterized their isolates further by examining phenotypic antibiotic resistance (using Kirby-Bauer or Sensititre^TM^ MIC panels) and by isolating native plasmids (Heringa et al., 2007). After short read Illumina sequencing, students in the course used the FDA’s GalaxyTrakr web platform for sequence trimming and filtering, assembly of draft genomes, sequence and genome assembly quality control, annotation of assemblies, and *in silico* serotyping. GalaxyTrakr is an instance of Galaxy^11^ that was developed by the FDA as a bioinformatics platform for use by United States public health laboratories. However, it is particularly advantageous for educational use, as the computational tools included are only those typically used for studying microbial genomes in general, particularly those of pathogens (Gangiredla et al., 2021). After they assembled, annotated, and serotyped their isolate genomes, students were then asked to pursue specific questions and hypotheses related to genes and gene functional categories of interest in their isolates, including those relating to antibiotic resistance, virulence, phages, plasmids, transposons, etc.

The 88 strains of *S. enterica* represented 19 unique serotypes. In the CDC’s report on the top 10 serotypes responsible for human infections in the United States^12^, five of the ten – Typhimurium, Infantis, Muenchen, Montevideo, and Braenderup – were repeatedly isolated in this study. Interestingly, a notable serotype that was not isolated was Enteritidis. This was surprising because Enteritidis is one of the most common serotypes associated with poultry^13^, yet no samples near or even directly from a poultry farm yielded this serotype.

Enterobase was used to identify 75 distinct core genome multilocus sequence types (cgMLSTs) among the 88 isolates (Table 1 and Figure 2). The cgMLST implemented in Enterobase is a highly discriminatory typing scheme. It reflects a so-called “soft-core genome” which in *Salmonella* consists of 3,002 genes found in ≥98%, intact in ≥94% and of “unexceptional diversity” in over 3,000 *Salmonella* genomes (Alikhan et al., 2018). A single base-pair difference in any of these genes would result in a different cgMLST. Six Braenderup isolates of cgMLST 4601 were isolated at different times and from different portions of Cooks Creek, with three isolates found upstream and three isolated 9–11 months later from an area ca. 7.5 km downstream. Since all belonged to the same core genome sequence type, there was no detected divergence between the isolates (Figure 2), suggesting that they may have come from a common source. It is possible that members of the same population of *Salmonella* from the upstream site moved down to the downstream site, or that there were independent introductions of the same type, perhaps from poultry litter spread on fields within the watershed.

Five strains were further sequenced using the Oxford Nanopore MinION^TM^ and hybrid-assembled to yield complete or near-complete circular assemblies. Two of these plasmids, pHJ38 and pFHS-02, were annotated and found to have multiple ARGs and two types of pilus genes potentially facilitating conjugation under multiple environmental conditions (Figures 3, 4). Plasmid pFHS-02 was particularly notable as it was a very large (303 kb) megaplasmid containing 11 predicted ARGs, 11 predicted heavy metal (mercury, arsenic, tellurite, and molybdenum) resistance genes, four predicted toxin-antitoxin systems, multiple transposons and IS elements, and a phage-associated virulence gene (*gipA*) associated with Peyer’s patch colonization and macrophage survival (Stanley et al., 2000; Vazeille et al., 2016). We have previously isolated numerous large, self-transmissible multidrug-resistant plasmids from many of these streams (Herrick et al., 2014).

We present here a model laboratory course design for introducing upper level microbiology undergraduates to real-world public health and pathogen surveillance methods and applications, as well as to laboratory research techniques and computational biology methods. At our university, this elective one-semester course is offered to students who have taken a course in general microbiology, including laboratory. Most of these students are majors in biology, typically with a concentration in microbiology, although some are allied health students majoring in health science, nursing, etc.

The course is divided into separate modules – one on the wet lab methods used for *Salmonella* isolation, isolation, and identification, and the other covering bioinformatics techniques. Linking the two modules is the *gratis* sequencing provided, in our case, by the United States FDA and our state public health laboratory, the Virginia DCLS. Foodborne and other related pathogens are of interest to many regional, state, and national public health laboratories for genomic epidemiological surveillance and these agencies are often willing, even eager, to sequence these at no cost. Although Module 1, focusing on the isolation of *Salmonella*, requires knowledge and skills in general microbiology laboratory techniques, the computational methods of Module 2 could conceivably be utilized by students with only a background in general college biology concepts. Module 2 in particular can be employed as a standalone research experience. Students could download the raw reads of *Salmonella* or other pathogens that are readily available from the NCBI Sequence Read Archive and work with them using the Bioinformatics Lab Guides available for this course^2^.

*Salmonella enterica* is one of the leading causes of foodborne illness in the world (Majowicz et al., 2010). It is also one of the most-sequenced organisms on earth^2^, due primarily to the massive efforts of agencies like the United States Food and Drug Administration and individual United States state public health laboratories. These and other national and regional agencies are interested in tracking the occurrence and spread of *S. enterica* and other pathogenic bacteria (Allard et al., 2018) and therefore are often willing to sequence, at no cost, the whole genomes of isolated strains, especially those isolated from less-sampled sources. However, public health agencies’ interest in the huge number of *Salmonella*, *E. coli*, Clostridioides, *Vibrio*, and other pathogens being sequenced worldwide rarely extends beyond cataloging their genomes for possible future epidemiological use. There is therefore a vast repository of essentially unanalyzed raw sequencing reads that have never been analyzed beyond a simple automated assembly and annotation, let alone examined for important accessory and other genetic elements such as plasmids, phages, transposons, ARGs, CRISPR regions, or virulence genes. This opens up an opportunity for students to work on authentic and important problems not only in genomic epidemiology and pathogen surveillance but also in mobile gene transfer, antibiotic resistance, the evolution of virulence, microbial ecology, etc. Students can potentially use and analyze either (1) strains they isolate themselves, or (2) the archived pathogen genomes in NCBI and other genome repositories. Depending on their needs, instructors could deploy a course such as this as a whole or as separate modules, one focusing on the wet lab isolation, identification, and characterization of *Salmonella* and the other on the genomics of *Salmonella* or other pathogens. Although *Salmonella* is a Biosafety II level (BSL 2) pathogen, it is relatively safe to work with in a classroom setting. It is not uncommonly cultivated for use in upper-division and even general microbiology laboratory courses (Ponder and Sumner, 2009; Marvasi et al., 2015). We have recently published details on safely setting up and using either module or the course in its entirety (Jurgensen et al., in press). We have also established an OSF page containing protocols, bioinformatics guides, safety documents, posters, etc. related to the course (see text footnote 1).

Over three-quarters of the students who took the course indicated they developed increased interest in research. Over 40% were interested in pursuing further study or research in bioinformatics and genomics and more than a third said that it influenced their future academic or career plans, particularly in relation to pathogen genomics and genomic epidemiology. Students in the course also benefited from their interaction with our research lab, where we are seeking to understand more specifically the ecology of the *Enterobacteriaceae* in secondary habitats such as streams, as well as their evolution via horizontal gene transfer. This course serves as a “feeder” of both data and interested students to more advanced research projects. Graduate and advanced undergraduate students from our research lab also serve as mentors to students in the course, especially with the more advanced aspects of their projects. Data generated from the course has been presented in a regional symposium^14^ and used in thesis projects (Jurgensen, 2018; Greenman, 2019).

This course provided a unique opportunity for microbiology students to gain valuable skills in pathogen isolation and identification, and in the basics of WGS and microbial genomics. Students were also introduced to the applications of these methods in public health microbiology, genomic epidemiology, pathogen surveillance, and in genome research in general. Course-based research experiences such as this can provide many of the benefits of traditional mentor-guided, open-ended and authentic research to students who might not otherwise have such an opportunity. They can also provide opportunities for students who have the interest to pursue further and deeper research questions on their isolates and their genomes, questions that are directly applicable to genomic epidemiology and to understanding the genomics and ecology of foodborne pathogens in the environment.

## Data Availability Statement

Illumina raw sequencing reads were deposited in the NCBI Sequence Read Archive (SRA) under BioProject PRJNA186035 for isolates HJ-01 through HJ-23 and BioProject PRJNA219491 for all others. Nanopore sequences for isolates HJ-21, HJ-24, HJ-29, HJ-38, and FHS-02 have been deposited in the SRA under BioProject PRJNA605356. SRR identifiers can be found in Table 1.

## Ethics Statement

Procedures involved in the collection and analysis of student data to assess the efficacy and impact of the research course, including the informed consent process and confidentiality parameters, were reviewed and approved by the James Madison University’s Institutional Review Board (IRB No. 18-0508).

## Author Contributions

JBH conceived the project. NAG, SKJ, CPH, CJK, and JBH designed the experiments. SKJ, CPH, CJK, RED, WMM, and JBH developed the methods. SKJ, RED, WMM, and JBH developed the instructional aspects of the *Bacterial Discovery* course. NAG, SKJ, CPH, CJK, RED, WMM, DE, BAE, SMH, TNK, HGL, DJL, and BW performed the experiments and conducted bioinformatics data analysis (undergraduates DE, BAE, TNK, HGL, DJL, and BW carried these out during the course itself). JAH gathered, compiled, and analyzed student assessment data. NAG, JBH, and JAH wrote the manuscript. SKJ, CJK, and RED edited it. The figures and table were generated by NAG. JSK, CPH, RED, WMM, DE, BAE, SMH, TNK, HGL, DJL, and BW were undergraduates at the time this work was done, NAG and CJK were graduate students, and JAH and JBH were on the faculty at James Madison University. All authors have read and approved the final manuscript.

## Conflict of Interest

The authors declare that the research was conducted in the absence of any commercial or financial relationships that could be construed as a potential conflict of interest.

## Footnotes

1 https://enterobase.warwick.ac.uk/

2 https://osf.io/gxut3/

3 https://five.epicollect.net/

4 https://www.bioinformatics.babraham.ac.uk/projects/fastqc/

5 https://github.com/rrwick/Porechop

6 https://github.com/nanoporetech/qcat

7 https://github.com/tseemann/abricate

8 http://www.genomicepidemiology.org/

9 http://www.galaxytrakr.org

10 https://environmentalintegrity.org/reports/water-pollution-from-livestock-in-the-shenandoah-valley/

11 http://www.usegalaxy.org

12 https://www.cdc.gov/nationalsurveillance/pdfs/2016-Salmonella-report-508.pdf

13 https://www.fsis.usda.gov/wps/portal/fsis/topics/data-collection-and-reports/microbiology/annual-serotyping-reports

14 http://www.asmbranches.org/brva/uploads/2/1/5/8/21583328/2018_asmva_final_program.pdf
